# Decay in Teeth

**Published:** 1848-10

**Authors:** J. Lee

**Affiliations:** Camden, S. C.


### ARTICLE IV.

### *Decay in Teeth.* By J. Lee, M. D., of Camden, S. C.

The ordinary cause assigned in books, and it is a popular error, is, that juxta-position causes decay in teeth, and then follows, as a consequence, the false practice of filing round teeth, to prevent decay; often leaving them rough from the file, thereby inducing the very disease the operation was intended to prevent.

There is certainly but little knowledge existing concerning the causes of decay. I have frequently seen the approximal surfaces of the front teeth decayed, soon after their protrusion from the gums, and sometimes the front surfaces of the same teeth. I have seen them with furrows and ridges across the front of the enamel, the ridges hard, and rounded in the furrows, the enamel disorganized, white, soft and crumbly, soon falling out, requiring a gold stopping, which seldom succeeds in arresting the decay. This decay I have seen described as leaving a worm-eaten appearance. By strict inquiry of parents, I have found in several cases, the children had, earlier than their sixth year, suffered with fever, and by comparing their times of illness with the growth of their teeth, I have referred it to the disease, or perhaps to mercurial medicines then exhibited. This congenital defective organization, whether from paternity or disease, is a frequent cause of decay. In its early stages, scarcely perceptible, it is suffered sometimes to progress, (which it does rapidly in the growing tooth,) until it is impossible to plug successfully; for in the growing tooth of a child, if the pulp is exposed, it cannot be destroyed and filled, the tooth not having arrived at maturity. If its growth be stopped, it decays rapidly, while in the adult tooth, a plug will succeed, the pulp being destroyed.

Some persons are slow to believe that the teeth come forward with material defects in their structure, but observing dentists have seen a want of enamel in the deep indentations, there acting as receptacles for decomposing particles of food, and of acid saliva. The bone is dissolved, and this insidious decay is going on under the enamel, often with an opening not larger than a small pin-hole, and escaping detection except by examination with a steel point in the hands of a dentist, or when, (often unfortunately too late,) chewing some hard substance, the whole surface breaks in, followed by acute tooth-ache. This occurring before the twelfth year, as it frequently does in the six-year grinders, extraction is the only remedy. It is possible they might have been saved, but experience shows that it is best to extract, as the twelve-year grinders will close up, and in a great measure take their places. More room is also afforded to the cuspidati, which frequently are crowded out of their regular place. The six-year grinders are usually mistaken by parents for deciduous teeth; from this, and the insidious manner in which the decay goes on, you rarely meet with an adult who has not lost one or more of them, or, having them remain in his mouth, ought to lose them. If the practice of filling deciduous teeth with tin was more common, then these teeth would more certainly come under the notice of the dentist, and often small plugs of gold would save many valuable teeth. They are, however, too often neglected until twelve or sixteen years of age; then they begin to ache, and are often irremediable. Much of this may be avoided by the family dentist urging the importance of attending to the changing of children's teeth, and making a light charge for filling the temporary teeth with tin, thereby saving much infantile suffering, the decay of the temporary teeth being at times distressing. It is therefore the duty of parents to have the teeth of children, under twelve years, inspected by their dentist every six months at the longest, and at shorter periods of time if convenient, and for which inspection no charge should be made. Not only would many valuable teeth be saved by plugging in time, but mal-positions in their incipiency could easily be prevented, and without expense, which, when fully formed, would cause expense, pain, and often the loss of valuable teeth, to be artificially supplied in after life. The loss of a molar tooth is a matter of much greater importance than it is generally estimated. The infliction of pain in the extraction is trifling compared with the after effects. Not the least of these is the wearing away of the front teeth, the loss of one grinder speedily causing the loss of others, they having extra duty to perform. The loss of a single grinder changes the countenance, and where several are lost, the cheeks fall in, and the voice changes; hence, where a tooth is curable, it should be cured. If extraction is unavoidable, the vacuity, as soon as the alveolus is rounded off, should be supplied with an artificial grinder on gold plate, which, if properly done, causes no pain, and after a week's wearing, no inconvenience. I therefore do not hold with those who recommend when a tooth is drawn from one jaw, that the antagonist be drawn; neither do the teeth naturally shut tooth on tooth, but two are made to receive the impulse of one.

The decay in the approximal surfaces of the teeth occurs more frequently (both in front teeth and grinders) in those parts not actually in contact, rendering it probable that decomposing portions of food are a frequent cause of decay. When the teeth are crowded, so as to slide on each other, then the pressure causes gangrene, but the approximal edges of the teeth never decay from juxta-position. Pressure acts on the teeth as it does on other organized parts. Such as are intended to support it, can do so harmlessly; such as are not so constructed always suffer from it, if long continued. But we often see similar points in similar teeth, running through several members of the same family, decayed, and in parts not subject to any pressure, or the lodgment of anything that might be supposed to have an influence in decaying them; this I have attributed to congenital defect.

Decay in teeth is gangrene, and should be treated as such. Plugging is always successful when there is a complete line of separation between the sound and unsound portions of the tooth. If, when you are excavating, the decayed portions present a smooth and polished surface, you may calculate with certainty on complete success; but if it is whitish, filmy, and with difficulty separable from the tooth, it is not in a fit state for filling, and however well done, the disease will continue. When, as is the case with the molar teeth of children, there are several diseased points, and you cannot by inspection tell what will be the course of the decay, it is well to wait; but in waiting you must fill the openings, if any exist, with tin, and in a few months the others will be developed.

My practice has been to fill any adult tooth having strength enough, and not too sensitive for the operation. When sensitive, use any of the preparations for allaying the sensibility, or plug with moistened tin, trusting to its oxydation, to prepare the cavity in three or six months, for a permanent filling of gold. By this treatment I have had the pleasure of saving many valuable teeth in adults, and this after others had passed them by as incurable. It is not always necessary or desirable to destroy the nerve of a front tooth, when exposed in excavating for plugging, neither should it be rounded; a little dexterity will remove the decay, leaving it in its bed. This should not be bridged with lead, but several smooth folds of foil should be first carried before the instrument, to form a smooth covering, and then successive portions passed down on each side, uniting in the middle as an arch. The cavity being half filled, you then go on and fill as an original cavity, equally pressing all over, and polishing the surface, and such plugs will stand secure. They are sensible to cold and heat four or five days.

When a tooth is filled, but one metal should be used, and this should be gold or tin. The practice of bridging a nerve with lead, and then filling with gold, ridiculous as it is, has had its advocates among those who should have known better, and every dentist of a few years practice has been called upon to remove plugs put in on this false principle, and which is sometimes carried out to the three-fourths filling the cavity with lead, and then coating with gold. Moisture here surely gets in. A small galvanic apparatus is formed in the tooth, and the oxydable metal becomes a dark powder, the gold sinks in, and the tooth is ready for extraction or for another plug. I will give three or four cases.

Mr. C—— called at my office, stating that he had his teeth well filled in ———, but that they annoyed him very much. With a probe, I examined the plugs. Two of the largest sank under the pressure. After taking out a small scale of gold, the balance of the filling was oxyd of tin or lead. He had paid the price of gold fillings. I refilled them with gold, and they gave him no farther trouble.

Mr. C—— called at my residence on Sabbath morning. He had suffered the previous night with pain in the head and face; referred it to a large molar tooth; examined it; had one large plug in the chewing surface; plug looked perfect, and resisted the probe; he insisted on having the tooth extracted, but suffered me first to take out the filling; found it half gold, and half lead or tin; I refilled it with gold, gave him an anodyne, and in ten hours, he was completely relieved.

Mr. M——. For this gentleman I filled five teeth with gold, and one, a large molar, I filled with tin, requesting him to let it remain in, until we met again. Not seeing me as early as expected, he had the tooth filled by a dentist of some celebrity. Some months after, he called on me, saying that he was suffering much with his large molar. I examined it, but could see no fault in the filling. Like Mr. C., he wished it extracted; I proceeded as in that case; found the same kind of plugging; replugged with about twenty grains of gold, and saved his tooth. This case occurred about two years since. Mr. C's, about seven years.

Mrs. H——. I was called to see this lady some eight years ago. Her case was similar to the others; suffering with pain in the face. I removed from her mouth ten plugs, apparently gold, eight of which were half of their substance lead or tin.

Where the approximal surfaces of the teeth are decayed, it is necessary to make space to excavate, and in a majority of cases, the file is indispensable. In the front teeth, there are cases where the loss of substance would mar the beauty of the tooth, and often on the opposite side there is space enough, if the tooth was moved over. This can be readily done in a few hours or a few days, by inserting small slips of gum elastic between the teeth. This was first used by Dr. Dana, some seven or eight years ago. Whether the practice originated with him I know not.

To excavate, the operator should be supplied with a variety of bent instruments; their edges should be sharp, and bent at different angles; they should be inserted at the edges, and not in the centre of the decay, so as to turn it out in flakes. The pain of this part of the operation is thereby much diminished, and the excavation expedited. The external opening of the cavity should be very little smaller than the body of the cavity. In deep cavities, I should prefer that the size be the same. If there be irregularities in the cavity, they should be filled up first in packing. No more gold should be carried before the instrument than can be securely placed at each pressure of the packer, and after the cavity is filled up, a fine-edged packer should be passed over every part of the plug before burnishing; to search for soft places, if any there be; then burnish so as to appear solid.

To give a general rule applicable to all cases, for putting in the foil, is impossible. Cutting the foil in strips, folding in several thicknesses, and then commencing at one end of the strip. Successive duplications are inserted until the cavity is full; but the mode of inserting these duplications depends more upon the particular case, than is generally supposed. If the foil is put in firmly and dry, not crumbled by too frequent movements of the instruments on one point, or dragging it from one part of the cavity to another, well pressed in and burnished, the plug will succeed. I have met with one operator who fills the small cavities in the front teeth with single strips of No. 6 foil, cut about as broad as the cavity. I have tried it, and succeeded very well. For these small cavities I prefer No. 4 gold, of the manufacture of Charles Abbey, Philadelphia, (and here permit me to say that for eight or ten years past I have bought more than half the foil I have used, from Mr. Abbey, and never has he sent me an article that I did not esteem superior to any that I have elsewhere purchased.) For the back teeth, I use Abbey's No. 6 gold. I prefer it to the heavier numbers. It has body enough, and pliancy, so that it can be readily pressed into the irregularities of any cavity.

Enumerating some of the causes of failure, the plugs dropping out, or not securing the teeth from decay, will show what is to be done to plug well. The first and too common cause is not completely excavating the decomposed bone.

2d. Depending on a decomposing edge of enamel to hold the plug.

3d. Removing the decayed bone, and filling on a surface predisposed to decay, without any preparation of the cavity.

4th. Filling large cavities when there are fissures in the enamel, opening or to open into them.

5th. Making too great a difference between the external opening and the body of the cavity, the parts under the edges not being well packed, breaking in on pressure.

6th. Not equally filling the cavity ; leaving some edge soft and subject to breakage.

7th. Not filling up the cavity and making an even surface.

8th. In large cavities not packing the bottom well. The surface looks well, but in a few weeks the grinding surface sinks below the enamel, as in 7, leaving the tooth as subject to decay as if it had not been filled. This is easily done by carrying too much gold before the packer.

9th. Having inferior materials, such as low priced gold foil, which is acted on by the secretions of the mouth, and this more especially when there are large tin or amalgam plugs in the vicinity.

10th. Using instruments too large, so that the gold does not adapt itself to the irregularities of the cavity.

11th. Using two metals in the same cavity.

To close an article on decay and its cure, without adverting to the use of cements, would perhaps be treating the subject as it deserves ; but the extravagant encomiums passed upon them by advertisers, and the ease with which they are introduced, caused many persons to be humbugged by them; and, unfortunately, many dentists, who ought to have known better, have advocated their use.

The cement most frequently coming under my observation is the amalgam of zinc and mercury, or silver and mercury; this, put into the tooth of an adult, becomes hard on the surface, and deceives the patient; he believes his tooth is cured, but the filling soon leaves the sides of the cavity, and the sides soon break off and leave it a black mass of oxyd. The excuse now given by the dentist is, the filling has lasted longer than the tooth. This is the least evil from the vile composition: nervous irritation frequently follows from the absorption of oxyd of mercury, and then a tooth that might, under judicious treatment, have been saved, must be extracted—for the patient is suffering too much to submit to scraping out the tooth and plugging it with gold; moreover, his dentist told him, if this cement did not save it nothing would. The tooth is lost—this is a great evil, but there is still a greater. Some systems are peculiarly susceptible of the mercurial influence—salivation follows; suppose the dentist sent for, he attributes the salivation to a dose of calomel, taken some six months or a year back; the evil remains in the mouth, and several teeth are lost. I am not here drawing on the imagination, for I could cite cases in illustration. Yet this preparation has been vaunted before the American public, as a substitute for gold stoppings. So much has been proved respecting their injurious tendency, that those who continue their use must submit to be gored by one horn of a disagreeable dilemma—ignorance or recklessness. For, even where they appear to succeed, they blacken the tooth in which they are introduced. Too many opportunities are yet afforded of witnessing their bad effects; I always remove them from the mouths of patients with their consent, and uniformly find them, where apparently successful, consisting of a hard crust, lying on a black powdery substance.

